# Supplementary material for: Smooth Interpolating Curves with Local Control and Monotone Alternating Curvature
Source: Comput Graph Forum. 2022 Oct 6;41(5):25–38. doi: 10.1111/cgf.14600 (PMC9827861; doi:10.1111/cgf.14600)
Supplement: Supplementary file 1 — Supplement Material [file CGF-41-25-s001.zip › Local-Smooth-Interpolating-MonoCurvature/extern/clothoids/submodules/quarticRootsFlocke/docs/api-cpp/file_PolynomialRoots-Jenkins-Traub.cc.html]

File PolynomialRoots-Jenkins-Traub.cc — Quartic Roots v1.0.0

### Navigation

- index
- toc
- Quartic Roots »
- File PolynomialRoots-Jenkins-Traub.cc

# File PolynomialRoots-Jenkins-Traub.cc¶

Contents

- Definition (`PolynomialRoots-Jenkins-Traub.cc`)
- Includes
- Namespaces
- Functions

## Definition (`PolynomialRoots-Jenkins-Traub.cc`)¶

- Program Listing for File PolynomialRoots-Jenkins-Traub.cc

## Includes¶

- `PolynomialRoots.hh` (File PolynomialRoots.hh)
- `cctype`
- `cfloat`
- `cmath`
- `fstream`
- `iostream`
- `limits`

## Namespaces¶

- Namespace PolynomialRoots

## Functions¶

- Function PolynomialRoots::roots

### Quick search

### Table of Contents

- Quartic Roots Solver
- C++ API

«
hide menu

menu
sidebar
»

### Navigation

- index
- toc
- Quartic Roots »
- File PolynomialRoots-Jenkins-Traub.cc

© Copyright 2021, Enrico Bertolazzi.
Created using Sphinx 4.2.0.
